# Differences in clinical features and morphology between differentiated and undifferentiated gastric cancer after *Helicobacter pylori* eradication

**DOI:** 10.1371/journal.pone.0282341

**Published:** 2023-03-31

**Authors:** Masaaki Kodama, Kazuhiro Mizukami, Yuka Hirashita, Tadayoshi Okimoto, Yasuhiro Wada, Masahide Fukuda, Sotaro Ozaka, Yoko Kudo, Kanako Ito, Ryo Ogawa, Kazuhisa Okamoto, Kensuke Fukuda, Kazunari Murakami

**Affiliations:** 1 Department of Gastroenterology, Faculty of Medicine, Oita University, Yufu, Japan; 2 Faculty of Welfare and Health Science, Oita University, Oita, Japan; Hospital Guillermo Kaelin de la Fuente, PERU

## Abstract

**Background/Aims:**

Although undifferentiated gastric cancer (UGC) diagnosed after *Helicobacter pylori* eradication (HPE) carries a poor prognosis, characteristics of post-HPE UGC have not been evaluated in detail because of its low incidence. Therefore, we compared the clinicopathologic characteristics of UGC and differentiated gastric cancers (DGC) diagnosed after successful HPE.

**Methods:**

GC lesions from patients who had successfully completed HPE and who had undergone upper gastrointestinal endoscopy between January 2004 and March 2016 were analyzed. Tumors were divided into DGC and UGC groups. Clinicopathologic factors of background and tumor characteristics were compared using univariate and multiple logistic analyses.

**Results:**

A total of 129 tumors from 115 patients were evaluated; 113 tumors were in the DGC group and 16 in the UGC group. Depressed-type tumors (*P* = 0.024) and sub-submucosal invasion (*P*<0.001) were significantly higher in the UGC group. The UGC group had larger tumor diameters (25.9±7.3 mm) than the DGC group (13.2±10.2 mm) (*P*<0.001). Multivariate analysis showed that female sex (odds ratio [OR] 3.24, 95%CI:1.02–10.37; *P* = 0.047) and absent follow-up (OR 4.99, 95%CI:1.60–15.57; *P* = 0.006) were significant independent risk factors for UGC. The DGC group showed a gradually decreasing temporal trend by trend test (*P* = 0.015), while the UGC group showed a relatively constant incidence over time, although the number of cases was small.

**Conclusion:**

UGC was diagnosed even after long time spans following HPE, although the number of cases was small. Female sex, and especially absent follow-up, were risks for post-HPE UGC, suggesting that diligent long-term follow-up after HPE is essential.

## Introduction

*Helicobacter pylori* is a well-known etiologic agent of many gastric mucosal diseases, such as chronic active gastritis, gastric atrophy, intestinal metaplasia, and finally gastric cancer (GC) [1]. *H*. *pylori* is a most important carcinogen of GC. The World Health Organization’s International Agency for Research on Cancer Working Group (IARC) recognized that *H*. *pylori* causes almost 90% of non-cardia GC [2]. Recent advances in the diagnosis and treatment of *H*. *pylori* infection have reduced its prevalence and the consequent incidence and mortality of GC [3, 4]. However, GC is still the third leading cause of cancer mortality worldwide, and carries especially high attributable mortality rates in East Asian countries. Moreover, GC incidence has increased recently in young people in several countries [4].

*H*. *pylori* eradication (HPE) has significantly reduced GC incidence [5–7]. IARC estimated that HPE may reduce incident GC by 30–40% [2]. However, HPE cannot prevent GC completely. Because the incidence of GC is about 2% after HPE [8, 9], the growing denominator of patients undergoing HPE has raised concern regarding a potential increase of post-HPE GC cases. Therefore, careful surveillance for GC is necessary even after HPE.

GC is classified into differentiated (DGC) and undifferentiated (UGC) histologic types [10]. Although most post-HPE GC show DGC histology [11–13], a small number present as UGC [9, 14,15]. Consequently, UGC has not been evaluated in detail because of its low incidence [15]. Patients with UGC often present at advanced stages and experience rapid progression and poor outcomes compared to those with DGC [16, 17]. Therefore, the prevention and early diagnosis of UGC are essential to improve the prognosis of patients who undergo HPE. In this study, we compared the clinicopathological features of post-HPE DGC and post-HPE UGC to elucidate the pathogenesis and risk factors of UGC presenting after HPE.

## Methods

### Subjects

GC lesions from patients who had previously undergone successful HPE and who had undergone upper gastrointestinal endoscopy at Oita University Hospital, Arita Gastrointestinal Hospital between January 2004 and March 2016 were analyzed. Successful HPE was defined by negative results on all of the following assays: rapid urease test, histology, culture testing, and urea breath test. Patients were evaluated for *H*. *pylori* re-infection during follow-up; those with recurrent infection were excluded. During this period, endoscopically detected GC cases were excluded if they were *H*. *pylori* positive (urease test, culture, or histology, any one of which was positive), *H*. *pylori* uninfected (all of urease test, culture, histology, serum anti-*H*. *pylori* antibody, endoscopic atrophy were negative), or had an unknown history of eradication.

All GC patients were re-evaluated for *H*. *pylori* infections during follow-up; no re-infections were detected. The absence of *H*. *pylori* infection and successful eradication was also confirmed by urease test, culture, and histology at the development of GC. Post-HPE GC were defined as GC diagnosed after successful HPE. Thus, GC discovered within one year after HPE were also included. The study protocol was approved by an institutional review board of Oita University, Faculty of Medicine (2339). All study procedures were in accordance with the ethical standards of the responsible committees on human experimentation (institutional and national) and with the Helsinki Declaration of 1964 and later versions. Informed consent was obtained from all participants or their responsible decision-makers in the form of opt-out on the web-site. Those who rejected were excluded.

### Comparison between DGC and UGC after HPE

The Japanese Classification of Gastric Carcinoma [10] was used for histological classification. pap: papillary adenocarcinoma, tubular adenocarcinoma; (tub1: well-differentiated; tub2: moderately differentiated) were defined as DGC. poorly differentiated adenocarcinoma (por1: solid type, por2: non-solid type) and sig: signet-ring cell carcinoma were designated as UGC [10]. In this study, histological evaluation was performed by M.K. and M.F., who majored in pathology.

GC cases were divided into DGC and UDG groups; cases exhibiting partial UDG were classified in the UDG group.

Patients were assigned to the “follow-up” group if they received annual endoscopy after successful HPE, or if GC was detected within one year of the last endoscopy after successful HPE. Subjects for whom endoscopy was not performed until the discovery of post-HPE GC were assigned to the “absent follow-up” group. Clinicopathologic factors such as age, sex, and other variables were compared between both groups using univariate and multiple logistic analyses.

### Comparison by tumor invasion depth

Lesions were divided into two groups according to the depth of tumor invasion: the group M for tumors confined within the mucosa and the group SM for tumors exhibiting submucosal or deeper invasion. Clinicopathological factors were compared.

### Comparison of the number of occurrences by time since HPE and by intragastric site

**Fig 1:**
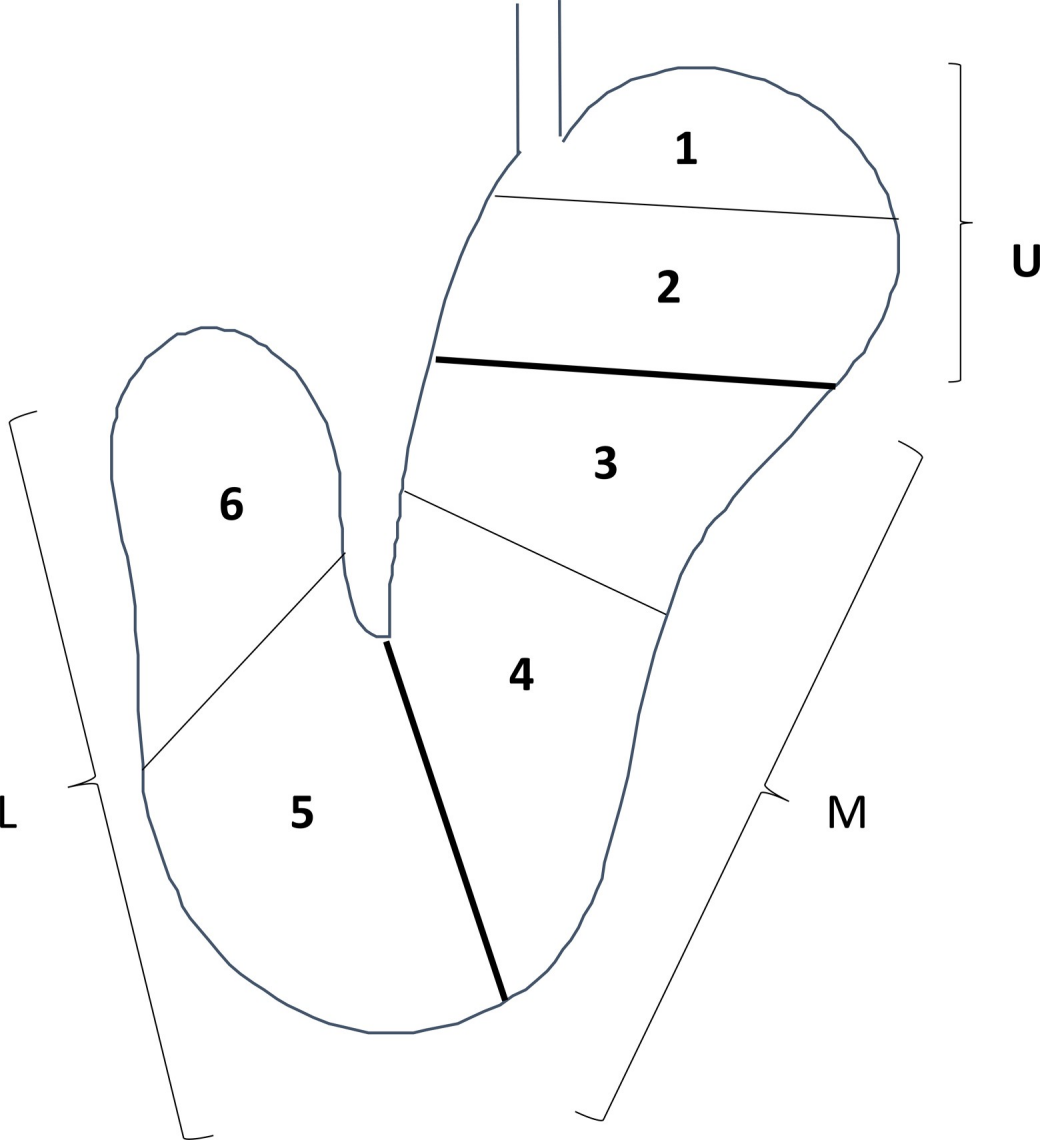
Three portions are defined by subdividing both the lesser and the greater curvatures into three equal lengths. U: Upper third, M: Middle third, L: Lower third; 1: Upper half of upper third. 2: Lower half of upper third, 3: Upper half of middle third, 4: Lower half of middle third, 5: Upper half of lower third, 6: Lower half of lower third.

DGC and UGC incidence rates were compared every 25 months after successful HPE. In addition, the stomach was divided into three portions, the upper (U), middle (M), and lower (L) parts, by the lines connecting the trisected points on the lesser and greater curvatures according to Japanese classification of gastric carcinoma (Fig 1). U, M, and L were further divided into two zones each, resulting in 6 zones to evaluate the site of GC occurrence (Fig 1). The association between the time from HPE to GC diagnosis and the number of GC occurrences in the DGC and UGC groups were compared.

### Endoscopic evaluation

Endoscopic atrophy was defined by using an endoscopic-atrophic-border scale reported by Kimura and Takemoto [18]. This scale correlates with histologic atrophy [19, 20]. Atrophy grades were also scored as C0: 0, C1: 1, C2: 2, C3: 3, O1: 4, O2: 5, and O3: 6, with 0 and 6 representing absent or severe atrophy, respectively.

### Statistical analysis

Statistical analyses were performed using SPSS software (SPSS Statistics 22, SPSS, Japan) and Microsoft Excel 2019 (Microsoft, USA). Data were expressed as mean ± standard deviation (SD). The Chi-square test and Fisher’s exact probability test were used to compare clinicopathological factors between the DGC and UGC groups. The Student T test was used to compare unpaired data, to perform a univariate analysis in terms of age, and tumor size. The Mann–Whitney *U* test was used to compare unpaired data, to perform a univariate analysis of in terms of endoscopic atrophy score. The Mantel-Haenszel test for trends was used to compare the trends in GC incidence. Multiplex logistic analysis was used for multivariable analysis to compare the clinicopathological factors of the DGC and UGC groups. Regression analysis and calculation of coefficient of determination (represented by R^2^) was performed to compare the anatomic sites of GC tumors and timespan after HPE. *P*-values < 0.05 were considered significant.

## Results

### Study sample

A total of 129 post-HPE GC lesions from 115 patients were evaluated (Table 1). One-hundred and 3 lesions occurred in males, and 26 developed in females. The mean age at HPE was 65.0 ± 8.8 years, mean age at GC diagnosis was 69.1 ± 9.4 years, and mean time from successful HPE to GC diagnosis was 54.1 ± 52.7 months. There were 88 primary and 41 metachronous GC, with patients developing up to 6 (one case) metachronous tumors (Table 1).

**Table 1:** Background of patients with gastric cancer following *H*. *pylori* eradication.

		GC after *H*. *pylori* eradication
Subjects (n)		115
Gastric cancers (n)		129
Primary, metachronous (2^nd^, 3^rd^, 4^th^, 5^th^, 6^th^) cancer		88/27/7/5/1/1
Sex (Male / Female)		103 / 26
Age at *H*. *pylori* eradication		65.0 ±8.52 y
Age at GC diagnosis		69.2 ±9.32 y
Duration between *H*. *pylori* eradication and GC diagnosis (months)		52.8±53.7
Background gastric diseases		
	Chronic gastritis	64
	Gastric ulcer (GU)	24
	Duodenal ulcer (DU)	5
	Gastroduodenal ulcer	1
	Gastric cancer	33
	Gastric adenoma	1
	MALT lymphoma	1
	GU / DU	24 / 5

### Clinicopathological differences between DGC and UGC groups

**Fig 2:**
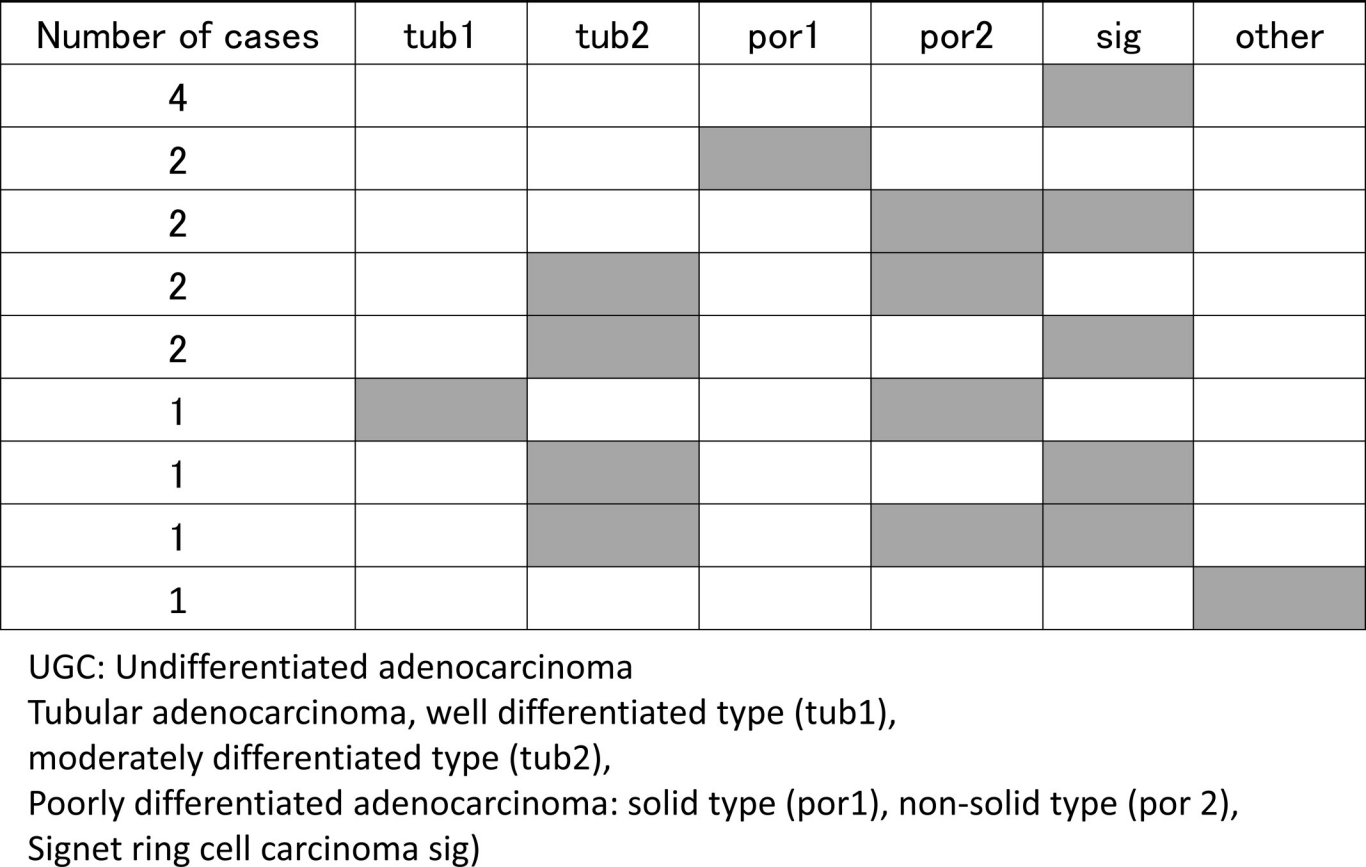
Distribution of intra-tumor histologic types of the UGC group. Several tumors featured different gastric cancer histologic types according to the Japanese Classification of Gastric Carcinoma (English version is 3rd 2011, October 2017).

There were 113 and 16 lesions in the DGC and UGC groups, respectively (Table 2). The incidence by U, M, L regions in the DGC group were 19, 50, and 44 cases; and 2, 7, and 7 cases in the UGC group, respectively (Table 2). There was no significant difference in incidence sites between two groups (*P* = 0.886). The proportion of depressed-type tumors was higher in the UGC group than in the DGC group (*P* = 0.024). Histologic classification disclosed 81 tub1 cases and 32 tub2 cases in the DGC group. The histologic distribution of cases in the UGC group revealed sig in only 4 cases and por1 in only 2 cases. Most of the other cases showed a mixture of histologic types (Fig 2). Tumor depth was M in 99 cases, SM in 12 cases, and MP in 2 cases in the DGC group; and M in 7 cases, SM in 5 cases, and MP in 4 cases in the UGC group. Sub-SM invasion was more prevalent in the UGC group than in the DGC group (*P*<0.001). Lymphovascular invasion rate was significant higher in UGC group than in DGC group (P = 0.003). The UGC group had significantly larger tumor diameters (25.9 ± 7.3 mm) than the DGC group (13.2 ± 10.2 mm) (*P*<0.001). All of patients showed no metastasis. As for treatment, significantly more lesions in the UGC group underwent surgery than ESD. There were no differences in the background diseases of DGC and UGC.

**Table 2:** Comparison between differentiated and undifferentiated adenocarcinoma after *H*. *pylori* eradication.

	Differentiated type	Undifferentiated type	*P*-value
Subjects (n)	99	16	-
Gastric cancers (n)	113	16	-
Location U/M/L	19 / 50 / 44	2 / 7 / 7	0.886^a^
Location U/M+L	19 / 94	2 / 14	0.710^a^
Macroscopic type	40/73	2/14	0.024^a^
Elevated/ depressed			
Histology	80 / 33 / 0	0 / 0 / 1	-
tub1/tub2/sig. por. Etc.			
Depth of invasion	99 / 12 / 2	7 / 5 / 4	<0.001
M/SM/ MP or deeper			
Depth of invasion	99 / 14	7 / 9	<0.001
M/ SM or deeper			
Lymphovascular invasion (+/-)	1/112	3/13	0.003^a^
Metastasis (+/-)	0/113	0/16	-
Cancer size (mm)	13.2 ± 10.2	25.9 ± 7.3	<0.001^b^
ESD / Surgery	110/3	6/10	<0.001^a^

ESD: Endoscopic submucosa dissection

Tumor size: Average ± SD

a: Statistical analysis was used chi-square test

b: Statistical analysis was used student’s t-test

### Comparison of background factors between DGC and UGC after HPE

Background factors of the DGC and UGC groups are compared in Table 3. Univariate analysis disclosed male/female ratios of 94/19 in the DGC group and 9/7 in the UGC group, with a significantly higher proportion of females in the UGC group (*P* = 0.019). The mean age at HPE was 65.5 ± 8.24 in DGC group and significantly lower (61.6 ± 9.34) in the UGC group (*P* = 0.045). There was no significant difference of age at GC diagnosis. There were more absent follow-up cases in the UGC group (8 of 16) than in the DGC group (27 of 113) (*P* = 0.0263). The degree of endoscopic atrophy at the time of HPE was 4.48 ± 1.40 in the DGC group and 4.67 ± 1.23 in the UGC group, showing no significant difference (*P* = 0.330).

**Table 3:** Univariate and multivariate analyses of differentiated and undifferentiated gastric cancer groups.

	Differentiated type GC	Undifferentiated type GC	Univariate analysis	Mulitivariate analysis		
			*P*-value	OR	95%CI	*P*-value
Subjects (n)	99	16	-			
Gastric cancers (n)	113	16	-			
Sex (Male/Female)	94 / 19	9/7	0.019^a^	3.24	1.02–10.37	0.047
Age at *H*. *pylori* eradication	65.5 ± 8.24	61.6 ± 9.34	0.045^b^			
Age at gastric cancer diagnosis	69.7 ± 8.96	66.0 ± 8.32	0.074^b^			
Follow up (+ / -)	86 / 27	8 / 8	0.0263^a^	4.99	1.60–15.57	0.006
Primary / Metachronous	75 / 38	13 / 3	0.240^a^			
History of gastric cancer						
Degree of endoscopic atrophy	4.48 ± 1.40	4.67 ± 1.23	0.330^b^			

GC: gastric cancer, Age, Degree of Endoscopic atrophy: Average ± SD

Statistical analysis was used chi-square test (a) and student’s t-test (b)

Multivariate analysis was used multiple logistic regression analysis. OR, odds ratio; CI, confidence interval.

Multivariate analysis using multiple logistic analysis showed that female sex and absent follow-up were significant independent risk factors, with odds ratios (OR) of female sex 3.24 (95%CI:1.02–10.37; *P* = 0.047) and absent follow-up 4.99 (95%CI:1.60–15.57; *P* = 0.006) (Table 3).

The male-female ratio of the degree of endoscopic atrophy in UGC was 4.29±1.25 for female and 4.67±1.32 for male (P = 0.284), and that of the degree of endoscopic atrophy in DGC was 4.71±1.69 for female and 4.43±1.34 for male (P = 0.232). No significant difference in endoscopic atrophy was found between males and females.

### Comparison of characteristics by GC invasion depth

Table 4 compares the groups with tumor invasion depths within M (M group) and those with SM or deeper invasion (SM group). There were significantly more cases with absent follow-up in the SM group (9 of 22) than in the M group (15 of 93) (*P* = 0.01), more cases in the M and L regions in the M group (82 of 93) than in the SM group (14 of 22) (*P* = 0.013), and more UGC in the SM group (8 of 22) than in the M group (6 of 93) (*P*<0.001). Tumor diameters were significantly larger in the SM group (22.3±16.5 mm) than in the M group (13.2±9.9 mm) (*P*<0.001).

**Table 4:** Comparison between gastric cancer with mucosal invasion and with deeper than submucosal invasion after *H*. *pylori* eradication.

	Group M (Invasion within M)	Group SM (Invasion deeper than SM)	*P*-value
Subjects (n)	93	22	-
Gastric cancers (n)	106	23	-
Sex (Male/Female)	76 / 19	19 / 4	0.85^a^
Age at *H*. *pylori* eradication	65.5 ± 8.2	62.8 ±10.7	0.09
Age at gastric cancer diagnosis	69.3 ± 9.1	67.9±10.5	0.27^b^
Follow up (+ / -)	78 / 15	13 / 9	0.01^a^
Degree of endoscopic atrophy	4.56±1.4	4.95±1.1	0.12^b^
Location U/M/L	13 / 44 / 38	8 / 9 / 5	0.035^a^
Location U/M+L	13 / 82	8 / 14	0.013^a^
Macroscopic type	40 / 66	6 / 16	0.69^a^
Elevated/ depressed			
Macroscopic type	21 / 32	1 / 10	0.052^a^
I/IIa/IIb/IIc			
Histology	65 / 24 / 6	6 / 8 / 8	<0.001^a^
tub1/tub2/sig. por. Etc.			
Depth of invasion	106 / 0 / 0 / 0	0 / 17 / 4 / 2	-
M/SM/MP/SE			
Cancer size (mm)	13.2±9.9	22.3±16.5	<0.001^a^

Age, tumor size: Average ± SD

a: Statistical analysis was used chi-square test

b: Statistical analysis was used student’s t-test

### Number of GC cases by time after HPE

**Fig 3:**
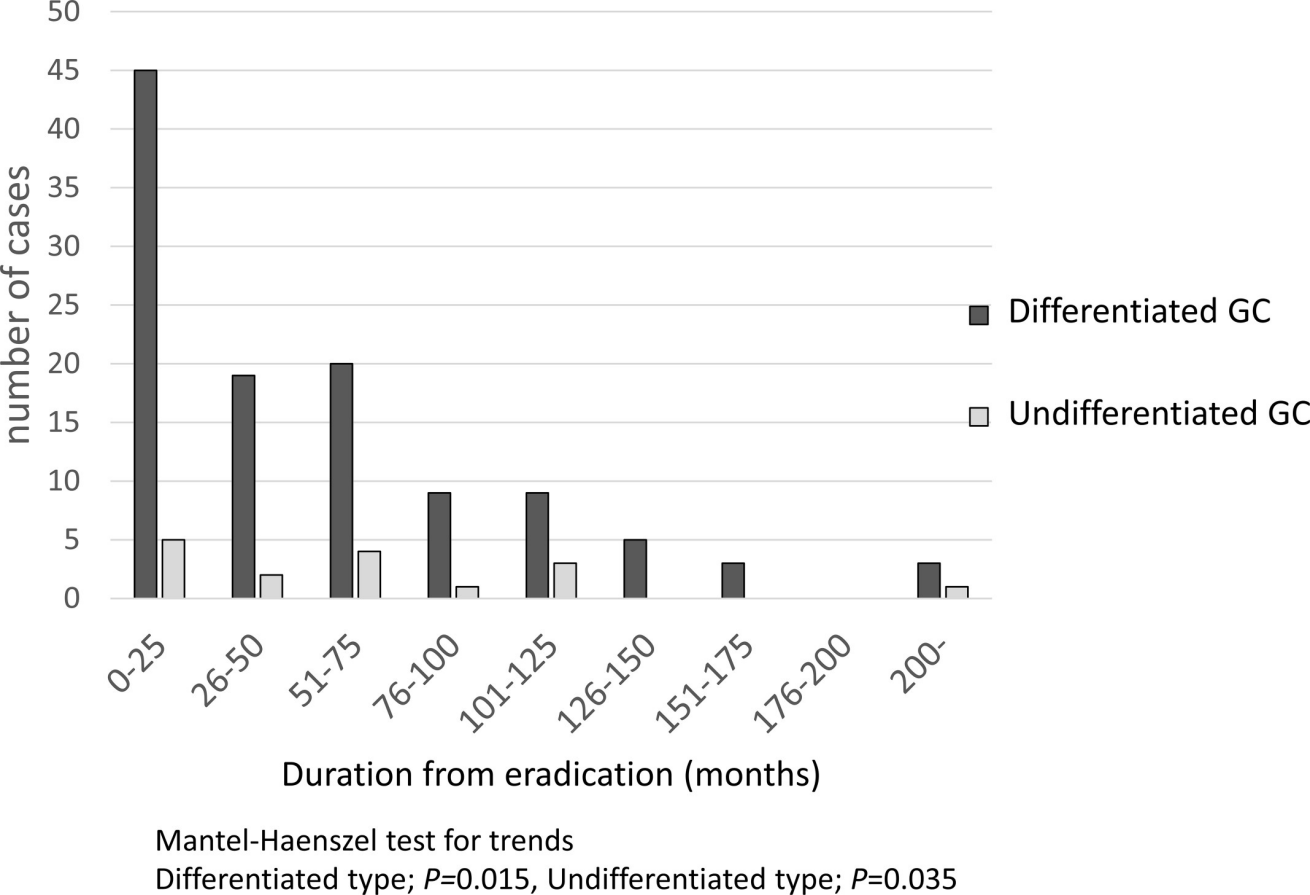
Number of gastric cancer incidence by time after eradication. Differentiated type gastric cancer showed a gradual decreasing trend (*P* = 0.015), while undifferentiated type gastric cancer showed the same frequency over a long time period, although the number of occurrences was small (*P* = 0.035).

Fig 3 shows the number of DGC and UGC cases according to time since HPE. The DGC group showed a gradually decreasing trend by trend test (*P* = 0.015), while the UGC group exhibited a relatively constant incidence over a long time span, although the number of occurrences was small (*P* = 0.035).

### Changes in GC incidence location according to post-HPE period

**Fig 4:**
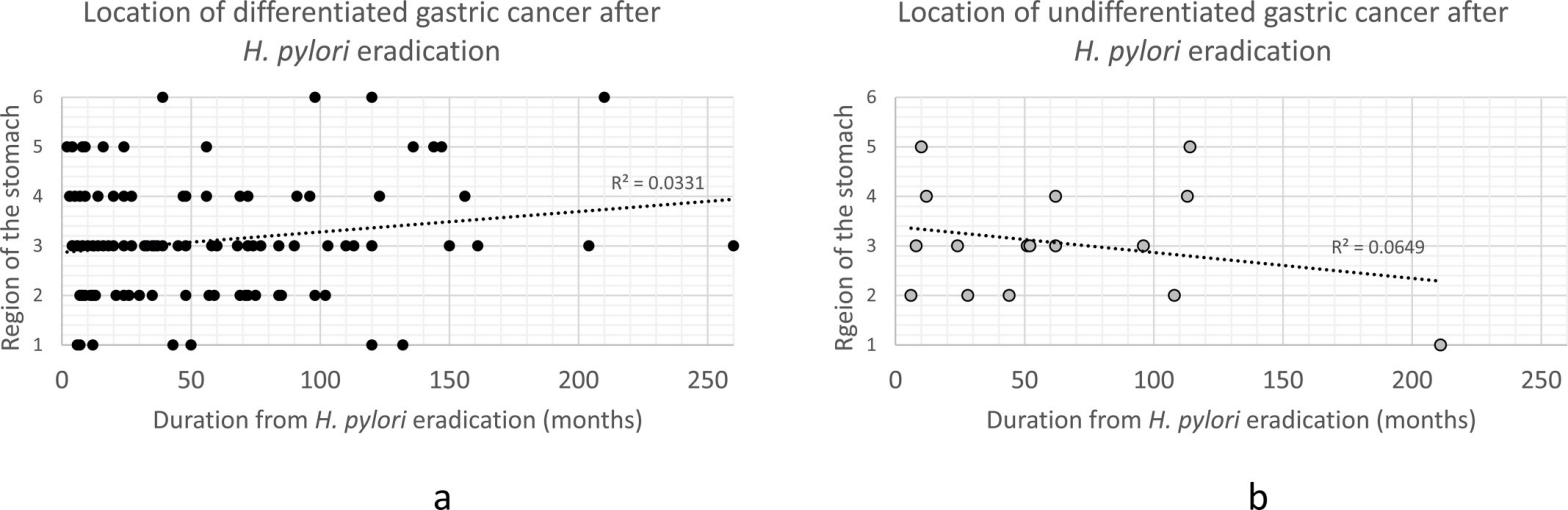
Gastric cancer incidence by time after eradication. Differentiated gastric cancer tended to occur on the U side (a), while undifferentiated gastric cancer tended to occur towards the L side after HPE (b). There was no significant trend in the location of gastric cancer over time in each group.

Fig 4 shows the relationship between the time from HPE to GC diagnosis and tumor location. DGC tumors tended to move toward the U side (Fig 4A), while UGC lesions tended to move toward the L side (Fig 4B), but R^2^ values (0.0331 and 0.0649, respectively), were low, and there was no significant trend in GC location over time.

## Discussion

In Japan, atrophy and intestinal metaplasia with *H*. *pylori*-infection are often severe, and the incidence of GC is high. Even after HPE, the development of GC is not completely controlled, therefore repeat endoscopic surveillance are performed. Jun et al [21]. reported that three or more screening endoscopy suppressed the mortality of GC. Management of epithelial precancerous conditions and lesions in the stomach (MAPS II) described that tighter endoscopy surveillance may be beneficial for the patients with extensive or incomplete intestinal metaplasia, persistent *H*. *pylori* infection, and the others [22]. However, caution should be exercised with UGC, which has a poor prognosis.

The proportion of GC diagnosed after HPE is increasing; furthermore, the characteristics of post-HPE GC are reportedly different from those of GC that develop during *H*. *pylori* infection [23–26]. Risk factors of post-HPE GC include male sex [8, 25], advanced age at HPE [14, 27], severe atrophy (endoscopic and histologic) [13, 14, 28–30], severe intestinal metaplasia [25, 31–34], and multiple GC before HPE [8]. Post-HPE GC is characterized by a predilection for the L region and antrum [28, 31, 32], small tumor size [11, 23], decreased tumor height [24], depressed-type morphology [23, 25], gastric-type mucosal phenotype [23, 25], and low grade epithelial atypia with “gastritis-like” appearance on endoscopy [26, 35]. However, most of these characteristics are features of post-HPE DGC.

Post-HPE GC often exhibits DGC histology [9, 23]. The incidence of post-HPE UGC is low, but not rare, even after long post-HPE time spans [14, 15]. Because UGC carries a poor prognosis, elucidating the characteristics of UGC after successful HPE is important. Although several studies have reported the features of post-HPE GC [23, 25], few have described the characteristics and carcinogenesis of post-HPE UGC. To our knowledge, this is the first report to compare the characteristics of post-HPE DGC and UGC and to examine the risk factors for post-HPE UGC.

The present study evaluated 113 DGC and 16 UGC tumors, with UGC having a much lower incidence than DGC; a constant ratio was observed for at least 125 months post-HPE. In several previous studies, UGC/DGC ratios ranged from 0.06 to 36.8% [11, 12, 14, 30]. Our results were within this range. Therefore, it is considered that the proportion of UGC development was consistent with previous studies.

The UGC group showed a significantly higher prevalence of SM invasion and lymphovasucular invasion than the DGC group, indicating that UGC is more likely to progress than DGC. The 5-year survival rate of cancer decreases as the T-factor or TNM stage increase. Lu et al. reported that in T2/T3 stage GC, UGC and UGC/DGC mixed-type tumors exhibited lower 5-year survival rates [36]. Furthermore, Song et al. found that DGC/UGC mixed-type tumors featured increased depth of invasion [37].

In addition, Tanaka et al. reported that UGC after HPE is similar to UGC before HPE, and that many UGC occurring more than 10 years after HPE showed submucosal and lymphovascular invasion [38]. In the present study, UGC/DGC mixed-type histology was also prevalent in the UGC group. Therefore, the rate of surgical resection was more higher than ESD. These findings highlight the necessity of early detection and treatment of UGC after HPE.

Multivariate analysis revealed that female sex and absent follow-up were independent risk factors, and that absent follow-up was the most significant risk factor for UGC. In contrast, the DGC group included a higher proportion of males. Female preponderance of UGC, both before and after HPE, has been reported previously [39]. Notably, nodular gastritis is a risk factor of UGC in young females [40, 41]. Vauhkonen et al. reported that although DGC occurred frequently in the setting of atrophic gastritis and intestinal metaplasia, UGC often arose in the absence of these abnormalities [42]. Gastric atrophy and/or intestinal metaplasia are more severe in males than in females [43, 44]; consequently, we suggest that the male preponderance of post-HPE DGC may be related to the differential sex-based severity of these predisposing conditions. Take also reported that UGC is more common in cases with mild gastric atrophy [14]. In this study, there was no difference in background atrophy between men and women for both DGC and UGC. This may be due to the fact that the comparison was only between tumors and not with the non-tumor group. However, the present results suggest that careful attention should be paid to the occurrence of DGC in males with severe gastric atrophy and UGC in females with mild gastric atrophy.

Cohort studies, randomized clinical trials, and meta-analyses have shown that HPE suppresses both GC in asymptomatic patients and metachronous recurrence after endoscopic resection of primary lesions [5, 6, 14, 45]. Because most post-HPE cases are DGC (larger denominator), HPE is more likely to reduce the number of DGC cases.

Takenaka et al. reported that HPE suppresses intestinal-type GC, but the suppression of diffuse-type GC is unclear due to the small number of UGC cases [15]. Furthermore, Take et al. reported that patients with mild to moderate gastric atrophy before HPE experienced a higher risk of UGC over post-HPE follow-up periods of up to 20 years [14]. The occurrence of UGC even after 200 months in this study suggests that the risk of UGC remains long after HPE, and that post-HPE endoscopic follow-up may be important.

In this study, the most significant independent risk factor of UGC was the absence of follow-up. Early GC diagnosis by regular endoscopic surveillance after HPE improves prognosis [46], which is consistent with our present results. Several reports have utilized assays of phenotypic mucin expression and genetic analysis to demonstrate that DGC transforms to UGC [47]. After evaluating differential mucin expression in early GC lesions, Saito suggested that small DGCs with gastric mucin-expressing phenotypes may transform into UGCs [47]. The loss of E-cadherin function in DGC may also be associated with progression to UGC [48]. In this study, GC that occurred in the absence of follow-up may have converted from DGC to UGC.

Our previous study indicated that gastric-type mucin was more prevalent than intestinal-type mucin in post-HPE GC [25]. In GC including UGC and papillary adenocarcinoma, gastric-type mucin expression has also been associated with aggressive neoplastic behavior, suggesting that post-HPE GC with gastric-type mucin may be more prone to UGC. The present results also show a significant increase in tumor diameter and a greater depth of invasion in UGC. These results suggest that during the absence of post-HPE follow-up, changes in gastric acid secretion [23], suppression of the internal mucin phenotype, and conversion from DGC to UGC may occur, thus promoting tumor growth and progression. Early detection of GC after HPE by diligent endoscopic follow-up is expected to reduce conversion to UGC and subsequent tumor progression.

Limitations of this study include the small sample size of patients with UGC. However, few reports have examined the clinicopathological features of post-HPE UGC; to our knowledge, the 16 UGC cases in our study comprise the largest number in studies that have examined UGC risk factors. Further studies should be performed with larger numbers of post-HPE DGC and UGC cases. The other limitation is that the study design did not distinguish between metachronous GC after GC treatment and primary GC without GC treatment. There is the difference in the incidence rate of primary and metachronous GC compared to that of primary GC [11, 14], and further study is needed with the increase the number of cases.

In conclusion, our study indicates that the clinicopathological characteristics of post-HPE UGC and DGC differ. UGC occurred even after longstanding HPE, although the number of cases was small. Female sex, and especially an absence of follow-up, were risk factors for post-HPE UGC, suggesting that diligent long-term follow-up after HPE is necessary.

## Supporting information

S1 Fig Histological findings of differentiated gastric cancer after *H*. *pylori* eradication. (TIFF)

S2 Fig Histological findings of undifferentiated gastric cancer after *H*. *pylori* eradication. (TIFF)

S1 File Clinicopathological data in all cases. (PDF)

S2 File (PDF)
