# Supplementary figures and images for: Differences in clinical features and morphology between differentiated and undifferentiated gastric cancer after Helicobacter pylori eradication
Source: PLoS One. 2023 Mar 31;18(3):e0282341. doi: 10.1371/journal.pone.0282341 (PMC10065271; doi:10.1371/journal.pone.0282341)

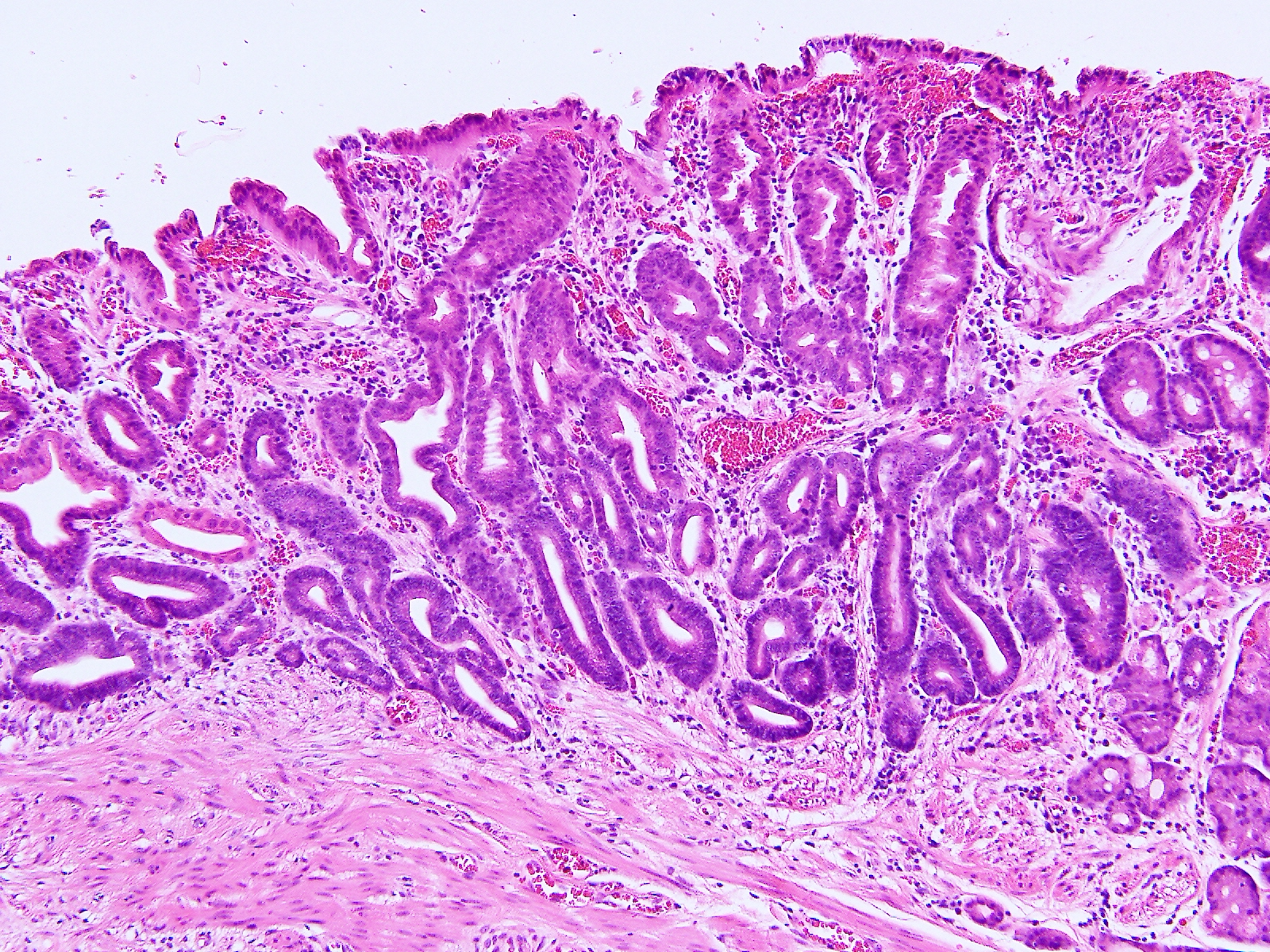

Supplement: S1 Fig — (TIFF) [file pone.0282341.s001.tiff]

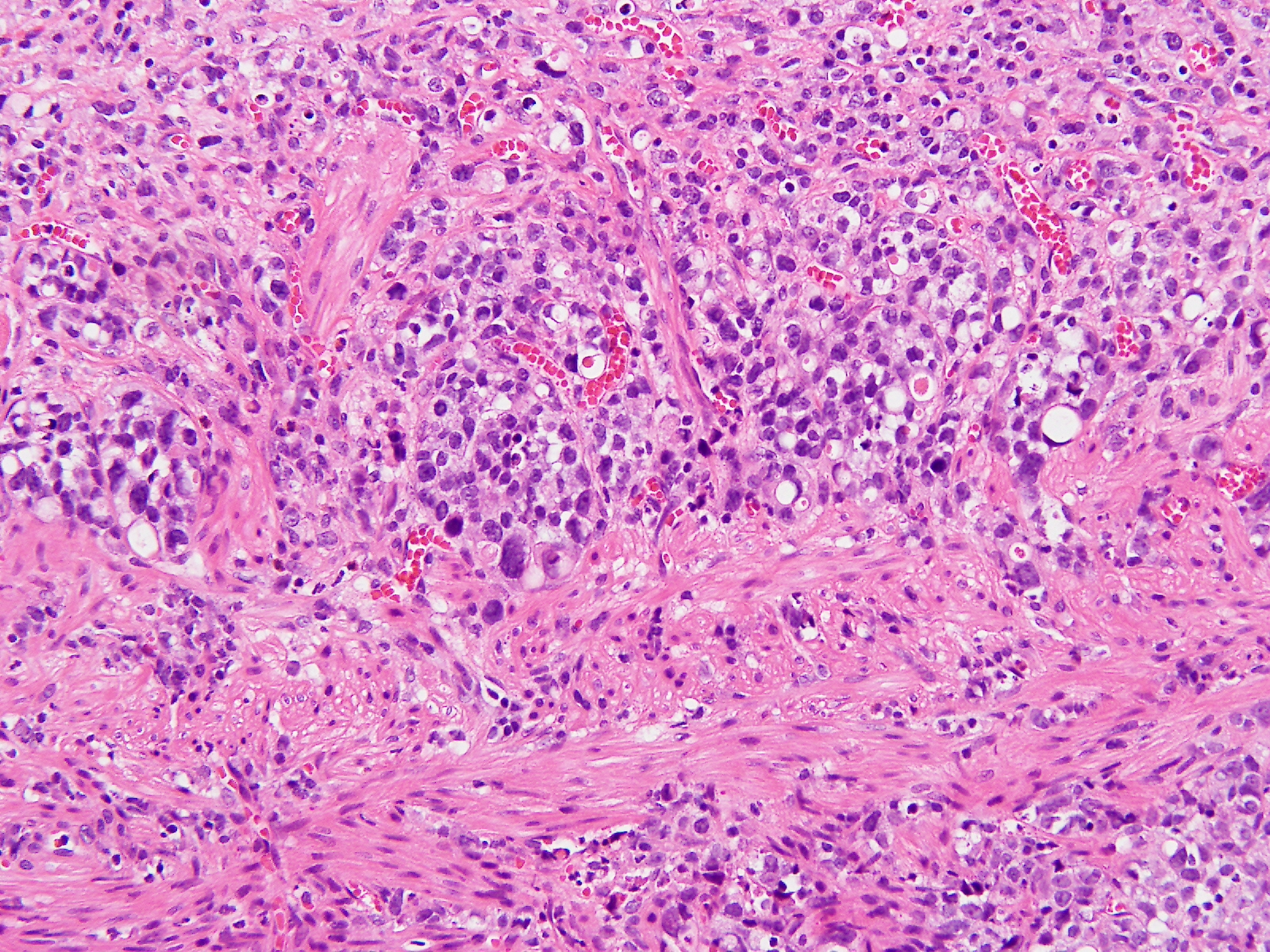

Supplement: S2 Fig — (TIFF) [file pone.0282341.s002.tiff]
