# Supplementary material for: Differences in clinical features and morphology between differentiated and undifferentiated gastric cancer after Helicobacter pylori eradication
Source: PLoS One. 2023 Mar 31;18(3):e0282341. doi: 10.1371/journal.pone.0282341 (PMC10065271; doi:10.1371/journal.pone.0282341)
Supplement: S1 File — (PDF) [file pone.0282341.s003.pdf]

No.	age at HPE	Sex	Duration after HPE	Follow up (+:1,-:0)	Location	Size1 (mm)	Size2 (mm)	Invasion	Histology (D:Differentiated, U:Undifferentiated)
1	65	F	123	1	U	15	15	M	D
2	65	M	33	1	M	10	10	M	D
3	61	F	18	1	M	20	20	M	D
4	70	M	113	1	M	20	7	M	D
5	57	F	16	1	M	25	8	M	D
6	63	M	59	1	M	10	6	M	D
7	60	M	12	1	L	5	5	M	D
8	70	M	26	1	L	20	5	M	D
9	63	M	57	0	L	10	8	SM	D
10	46	M	7	1	L	10	10	M	D
11	74	M	6	1	L	20	4	M	D
12	68	M	48	1	U	0.7	0.7	M	D
13	57	M	60	1	L	25	25	M	D
14	72	M	2	1	U	23	20	M	D
15	70	M	24	0	U	11	20	M	D
16	59	M	136	1	U	10	8	M	D
17	59	M	96	1	M	12	12	M	D
18	80	M	24	1	M	6	4	M	D
19	47	F	27	1	M	35	28	SE	D
20	70	M	47	1	U	15	6	M	D
21	68	M	3	0	U	31	31	M	D
22	61	F	56	0	U	10	10	M	D
23	71	F	69	1	M	19	11	M	D
24	64	M	5	1	U	9	8	SM	D
25	63	M	156	0	U	10	10	M	D
26	71	M	48	1	L	10	8	M	D
27	71	M	12	1	L	5	5	M	D
28	80	M	120	0	M	5	5	M	D
29	52	F	74	0	M	29	17	M	D
30	70	F	103	1	M	15	12	M	D
31	78	M	27	1	M	10	7	M	D
32	73	M	4	0	M	13	11	M	D
33	63	M	37	1	M	10	10	M	D
34	65	M	110	0	M	9	5	M	D
35	57	M	150	1	L	26	17	M	D
36	80	M	8	1	M	10	8	SM	D
37	64	M	58	0	M	13	13	M	D
38	54	F	6	1	M	31	31	M	D
39	56	M	36	0	M	14	14	M	D
40	59	M	120	1	M	7	7	M	D
41	52	F	85	0	L	15	13	M	D

42	52	F	30	0	L	25	23	M	D
43	68	M	7	1	L	20	18	M	D
44	65	M	8	1	L	5	5	M	D
45	57	M	24	0	L	12	12	M	D
46	72	M	9	1	L	14	11	M	D
47	59	M	8	1	L	20	15	M	D
48	68	M	98	0	L	12	12	M	D
49	74	M	12	0	L	12	12	M	D
50	75	M	7	0	L	5	7	M	D
51	62	F	39	1	U	7	4	M	D
52	54	M	210	0	U	22	13	SM	D
53	66	M	16	1	M	6	6	M	D
54	67	M	144	1	U	3	2	M	D
55	67	M	147	1	U	11	9	M	D
56	62	M	69	1	L	7	6	M	D
57	62	M	14	1	M	5	4	M	D
58	62	M	156	1	M	10	7	M	D
59	67	M	20	1	M	22	13	SM	D
60	68	M	72	1	M	4	4	M	D
61	83	F	72	1	M	12	7	M	D
62	68	M	68	0	L	55	50	M	D
63	59	M	39	1	M	5	4	M	D
64	58	F	24	1	M	7	4	M	D
65	73	M	72	1	M	6	5	M	D
66	65	F	90	1	M	5	4	M	D
67	60	m	260	1	L	13	9	M	D
68	67	M	20	1	M	7	4	M	D
69	68	M	72	1	M	8	5	M	D
70	65	m	6	1	M	1	1	M	D
71	74	F	14	1	L	16	7	M	D
72	73	M	72	1	L	16	10	M	D
73	76	M	102	0	L	15	12	M	D
74	62	M	69	1	L	10	8	M	D
75	51	m	12	1	L	40	35	M	D
76	68	M	75	1	L	7	4	M	D
77	73	M	21	1	L	7	6	M	D
78	63	M	84	1	L	11	10	M	D
79	62	M	120	1	L	5	4	M	D
80	67	M	132	1	L	6	4	M	D
81	55	M	91	0	M	11	18	M	D
82	51	M	84	1	M	14	14	SM	D
83	48	M	32	1	M	10	6	M	D
84	75	M	4	1	U	10	3	M	D
85	53	M	13	1	L	20	20	M	D
86	71	M	48	1	L	15	15	M	D
87	66	M	120	0	U	21	30	M	D
88	73	M	98	1	U	28	22	MP	D
89	70	M	9	1	U	15	8	SM	D

90	76	M	4	1	U	76	40	SM	D
91	68	M	7	1	M	11	20	M	D
92	74	M	4	0	M	15	15	M	D
93	80	M	60	1	M	20	15	SM	D
94	52	M	161	1	M	27	22	M	D
95	66	F	35	1	M	21	30	M	D
96	75	M	204	0	M	21	30	M	D
97	45	M	12	1	L	15	10	M	D
98	64	M	43	0	L	5	7	M	D
99	75	F	50	0	L	12	12	M	D
100	62	M	8	1	M	7	5	M	D
101	67	M	56	1	U	16	4	SM	D
102	62	M	9	1	M	7	5	M	D
103	69	M	20	1	M	19	14	M	D
104	73	M	10	1	M	11	8	M	D
105	78	M	45	0	L	8	8	SM	D
106	76	F	60	1	L	16	8	M	D
107	68	M	77	1	M	6	6	M	D
108	65	m	6	1	M	16	10	M	D
109	78	M	35	1	L	10	6	M	D
110	68	M	71	1	L	55	50	M	D
111	68	M	26	1	L	11	10	M	D
112	51	m	11	1	M	9	5	SM	D
113	69	M	24	1	L	10	7	M	D
114	61	F	10	1	U	55	55	MP	U
115	64	M	114	1	U	30	30	MP	U
116	51	F	62	0	M	20	15	SM	U
117	53	M	51	0	M	35	35	MP	U
118	67	M	44	0	L	38	32	M	U
119	72	M	52	1	L	15	15	SM	U
120	61	F	96	0	L	22	10	M	U
121	60	M	108	0	L	15	8	SM	U
122	55	M	113	0	M	30	13	SM	U
123	51	F	62	1	M	8	6	M	U
124	52	F	6	1	L	20	20	SM	U
125	81	M	8	0	M	12	12	M	U
126	66	F	24	0	M	11	11	M	U
127	52	M	211	1	L	68	39	SE	U
128	63	M	12	1	M	66	50	M	U
129	77	F	28	1	L	19	14	M	U
