# Supplementary material for: Differences in clinical features and morphology between differentiated and undifferentiated gastric cancer after Helicobacter pylori eradication
Source: PLoS One. 2023 Mar 31;18(3):e0282341. doi: 10.1371/journal.pone.0282341 (PMC10065271; doi:10.1371/journal.pone.0282341)
Supplement: S2 File — (PDF) [file pone.0282341.s004.pdf]

No.	除菌後期間	location	Histology
1	123	U	Differentited
2	33	M	Differentited
3	18	M	Differentited
4	113	M	Differentited
5	16	M	Differentited
6	59	M	Differentited
7	12	L	Differentited
8	26	L	Differentited
9	57	L	Differentited
10	7	L	Differentited
11	6	L	Differentited
12	48	U	Differentited
13	60	L	Differentited
14	2	U	Differentited
15	24	U	Differentited
16	136	U	Differentited
17	96	M	Differentited
18	24	M	Differentited
19	27	M	Differentited
20	47	U	Differentited
21	3	U	Differentited
22	56	U	Differentited
23	69	M	Differentited
24	5	U	Differentited
25	156	U	Differentited
26	48	L	Differentited
27	12	L	Differentited
28	120	M	Differentited
29	74	M	Differentited
30	103	M	Differentited
31	27	M	Differentited
32	4	M	Differentited
33	37	M	Differentited
34	110	M	Differentited
35	150	L	Differentited
36	8	M	Differentited
37	58	M	Differentited

38	6	M	Differentited
39	36	M	Differentited
40	120	M	Differentited
41	85	L	Differentited
42	30	L	Differentited
43	7	L	Differentited
44	8	L	Differentited
45	24	L	Differentited
46	9	L	Differentited
47	8	L	Differentited
48	98	L	Differentited
49	12	L	Differentited
50	7	L	Differentited
51	39	U	Differentited
52	210	U	Differentited
53	16	M	Differentited
54	144	U	Differentited
55	147	U	Differentited
56	69	L	Differentited
57	14	M	Differentited
58	156	M	Differentited
59	20	M	Differentited
60	72	M	Differentited
61	72	M	Differentited
62	68	L	Differentited
63	39	M	Differentited
64	24	M	Differentited
65	72	M	Differentited
66	90	M	Differentited
67	260	L	Differentited
68	20	M	Differentited
69	72	M	Differentited
70	6	M	Differentited
71	14	L	Differentited
72	72	L	Differentited
73	102	L	Differentited
74	69	L	Differentited
75	12	L	Differentited
76	75	L	Differentited
77	21	L	Differentited

78	84	L	Differentited
79	120	L	Differentited
80	132	L	Differentited
81	91	M	Differentited
82	84	M	Differentited
83	32	M	Differentited
84	4	U	Differentited
85	13	L	Differentited
86	48	L	Differentited
87	120	U	Differentited
88	98	U	Differentited
89	9	U	Differentited
90	4	U	Differentited
91	7	M	Differentited
92	4	M	Differentited
93	60	M	Differentited
94	161	M	Differentited
95	35	M	Differentited
96	204	M	Differentited
97	12	L	Differentited
98	43	L	Differentited
99	50	L	Differentited
100	8	M	Differentited
101	56	U	Differentited
102	9	M	Differentited
103	20	M	Differentited
104	10	M	Differentited
105	45	L	Differentited
106	60	L	Differentited
107	77	M	Differentited
108	6	M	Differentited
109	35	L	Differentited
110	71	L	Differentited
111	26	L	Differentited
112	11	M	Differentited
113	24	L	Differentited
114	10	U	undifferentiated
115	114	U	undifferentiated
116	62	M	undifferentiated
117	51	M	undifferentiated

118	44	L	undifferentiated
119	52	L	undifferentiated
120	96	L	undifferentiated
121	108	L	undifferentiated
122	113	M	undifferentiated
123	62	M	undifferentiated
124	6	L	undifferentiated
125	8	M	undifferentiated
126	24	M	undifferentiated
127	211	L	undifferentiated
128	12	M	undifferentiated
129	28	L	undifferentiated
